# Quantifying the link: coronary artery inflammation via CCTA-derived fat attenuation index and its association with diabetes duration

**DOI:** 10.3389/fendo.2025.1671949

**Published:** 2025-12-04

**Authors:** Yan Zhang, Jing Wang, Kexin Song, Zhuhua Yao

**Affiliations:** 1 Department of Cardiology, Tianjin Union Medical Center, The First Affiliated Hospital of Nankai University, Tianjin, China; 2 The Institute of Translational Medicine, Tianjin Union Medical Center of Nankai University, Tianjin, China; 3 Department of Cardiology, Tianjin Medical University Chu Hsien-I Memorial Hospital, Tianjin, China

**Keywords:** cardiovascular disease, high fasting plasma glucose, pericoronary fat attenuation index, coronary artery inflammation, diabetes duration

## Abstract

**Aims:**

Although diabetes is a well-established enhancer of coronary inflammation, the specific relationship between diabetes duration and the degree of inflammation, as quantified by pericoronary fat attenuation index (FAI), remains poorly defined. This study aimed to investigate the association between diabetes duration and coronary inflammation, as assessed non-invasively by the pericoronary FAI derived from coronary computed tomography angiography (CCTA).

**Materials and methods:**

We enrolled 468 adults with type 2 diabetes mellitus who underwent CCTA imaging. The pericoronary FAI was quantified around the three main coronary arteries. Multivariable linear regression and subgroup analyses were performed to evaluate the association between diabetes duration and pericoronary FAI. Potential non-linear associations were examined using restricted cubic spline (RCS) modelling.

**Results:**

Longer diabetes duration was independently and positively associated with increased pericoronary FAI values in the LAD artery (β = 0.151, 95% CI: 0.064–0.238, P = 0.001), LCX artery (β = 0.101, 95% CI: 0.001–0.201, P = 0.047), and RCA (β = 0.208, 95% CI: 0.120–0.296, P < 0.001). RCS modelling revealed predominantly linear associations(P for non-linearity > 0.05). The association between diabetes duration and pericoronary FAI remained robust across the majority of examined subgroups.

**Conclusion:**

Prolonged diabetes duration is independently associated with elevated coronary inflammation, demonstrating a near-linear dose-response relationship.

## 1 Introduction

Cardiovascular disease (CVD) remains the leading global cause of mortality and disability, exerting a substantial and growing burden on healthcare systems worldwide (1, 2). Type 2 diabetes mellitus (T2DM) is a powerful, modifiable, and independent risk factor that markedly accelerates the onset and progression of atherosclerosis and its downstream complications, including myocardial infarction and stroke (3, 4). Although the association between diabetes and CVD is well established, key knowledge gaps remain regarding the underlying contributors to excess cardiovascular risk at individual pathophysiological levels.

Diabetes duration is increasingly recognized as a pivotal determinant of cumulative vascular injury (5–7). Prolonged diabetes duration is strongly correlated with chronic hyperglycemic exposure and the accumulation of additional cardiovascular risk factors (8–10).Vascular remodeling represents a pivotal component in the pathogenesis of atherosclerosis and CVD (11, 12). Emerging evidence highlights the critical role of microRNAs (miRNAs) as key post-transcriptional regulators in vascular remodeling (13). Dysregulation of miRNAs under hyperglycemic conditions may potentiate vascular remodeling and accelerate atherosclerotic progression, thereby providing a mechanistic link between the duration of diabetes and coronary artery inflammation (14, 15). Coronary artery inflammation represents a central pathological mechanism underlying CVD, driving both plaque vulnerability and atherosclerotic progression (16–19). Pericoronary adipose tissue (PCAT), a metabolically active component of epicardial fat, envelops the coronary arteries (20). Owing to its direct anatomical proximity, PCAT facilitates bidirectional exchange of proinflammatory mediators with the coronary vasculature (21). Under hyperglycemic conditions, dysfunctional PCAT secretes proinflammatory cytokines into adjacent arterial walls, thereby accelerating atherogenesis (22, 23). Conversely, inflamed coronary arteries elicit phenotypic alterations in adjacent PCAT adipocytes, characterized by reduced lipid density and increased water content. These structural changes result in elevated computed tomography attenuation values (24, 25).As a result, the fat attenuation index (FAI), derived from coronary computed tomography angiography (CCTA), has emerged as a novel, reliable, and non-invasive imaging biomarker of coronary artery inflammation (20, 26, 27). Although T2DM is a well-established enhancer of coronary inflammation, the specific relationship between diabetes duration and the degree of inflammation, as quantified by pericoronary FAI, remains poorly defined. Clarifying this association is critical for cardiovascular risk stratification and for identifying patients most likely to benefit from targeted anti-inflammatory interventions.

This study aimed to investigate the relationship between diabetes duration and coronary artery inflammation—measured by perivascular FAI on CCTA—in a Chinese clinical cohort.

## 2 Materials and methods

### 2.1 Study population

This cross-sectional study conducted a retrospective screening of individuals diagnosed with T2DM who underwent CCTA at Tianjin Union Medical Center between January 2024 and June 2025. CCTA data from 641 enrolled patients with T2DM were initially analyzed, with 173 patients subsequently excluded due to missing medical record data (n = 16), insufficient image quality to calculate relevant data (n = 39), or history of prior CVD (n = 118). Final 468 eligible patients were included in the analysis. This study was approved by the Clinical Research Ethics Committee of Tianjin Union Medical Center and was conducted in accordance with the principles of the Helsinki Declaration. Due to the retrospective nature of the study, the informed consent exemption was approved by the Ethics Committee.

### 2.2 Data collection

The clinical and laboratory data of the enrolled cohort were meticulously extracted from the medical records database. Diabetes duration was defined as the time interval between the date of first clinical diagnosis of T2DM and the date of CCTA examination. Patients who were actively smoking or had quit smoking within the past year were classified as smoking. BMI was calculated as weight in kilograms divided by height in meters squared (kg/m²).Hypertension was defined as systolic blood press ≥140 mmHg and/or diastolic blood press ≥90 mmHg, or the use of antihypertensive medication (28). Dyslipidemia was defined as total cholesterol ≥6.2 mmol/L, triglycerides ≥2.3 mmol/L, low-density lipoprotein cholesterol(LDL-C) ≥4.1 mmol/L, high-density lipoprotein cholesterol(HDL-C) <1.0 mmol/L, or current lipid-lowering treatment (29).

CCTA was performed using a 320-slice scanner (Aquilion ONE; Canon Medical Systems, Japan).Scan coverage extended from approximately 1 cm below the tracheal carina to the diaphragm of the heart. Acquisition parameters were tube voltage (100 or 120 kV) and tube current (200–600 mA), adjusted according to BMI. Patients with baseline heart rate >75 bpm received oral metoprolol. All scans employed a single breath-hold technique to ensure diagnostic image quality. PCAT attenuation analysis was conducted using semi-automated software (CoronaryDoc, Shukun Technology, China) following established protocols. PCAT was characterized as adipose voxels (−190 to −30 HU) within a radial distance from the coronary vessel wall equal to the vessel diameter. Measurements were obtained in the proximal 40-mm segments of the left anterior descending (LAD) and left circumflex (LCX) arteries, and the proximal 10–50 mm segment of the right coronary artery (RCA) (30). Mean attenuation values of perivascular adipose tissue were used for PCAT quantification.

### 2.3 Statistical analysis

For analytical purposes, participants were stratified into two groups based on diabetes duration: ≤10 years versus >10 years. The 10-year threshold was determined *a priori*, informed by prior studies indicating a pronounced acceleration in cardiovascular risk (31, 32) Normally distributed continuous variables were presented as mean ± standard deviation and compared using the t-test. Non-normally distributed continuous variables were expressed as median (interquartile range) and compared using the Mann-Whitney U test. Categorical data were described as frequencies (percentages) and compared using chi-square test. Bivariate associations between diabetes duration and traditional CVD risk factors were examined using Spearman correlation analysis. The correlation between the diabetes duration and pericoronary FAI was evaluated using multivariate linear regression analysis across three distinct models. The selection of covariates was guided by both known factors (33, 34) and statistical considerations. Stepwise regression analysis were conducted to identify independent predictors. Model 1 was not adjusted for covariates. Model 2 adjusted for age, gender, and BMI. Model 3 further adjusted for dyslipidemia, smoking, antihyperlipidemic agents, antidiabetic agents, CACS, LVEF, and HbA1c. Multicollinearity was assessed using variance inflation factors (VIFs). None of the variables demonstrated a VIF exceeding 5, indicating the absence of substantial multicollinearity (**Supplementary Table S1**). The association between the diabetes duration and pericoronary FAI was assessed in the models using coefficients (β) and 95% confidence intervals (CI). Restricted cubic spline (RCS) models, adjusted for Model 3 covariates, were used to explore potential non-linear associations between diabetes duration and pericoronary FAI. Subgroup analysis was performed using multivariate linear regression (model 3) stratified by gender, age, BMI, smoking status, hypertension, and dyslipidemia. To address potential confounding due to medication use, a sensitivity analysis was performed with adjustment for specific antihyperlipidemic and antidiabetic agents. All statistical analyses were conducted using SPSS version 25.0 (IBM Corp., Armonk, NY, USA) and R version 4.5. A two-sided P-value < 0.05 was considered statistically significant.

## 3 Results

### 3.1 Baseline characteristics

**Figure 1:**
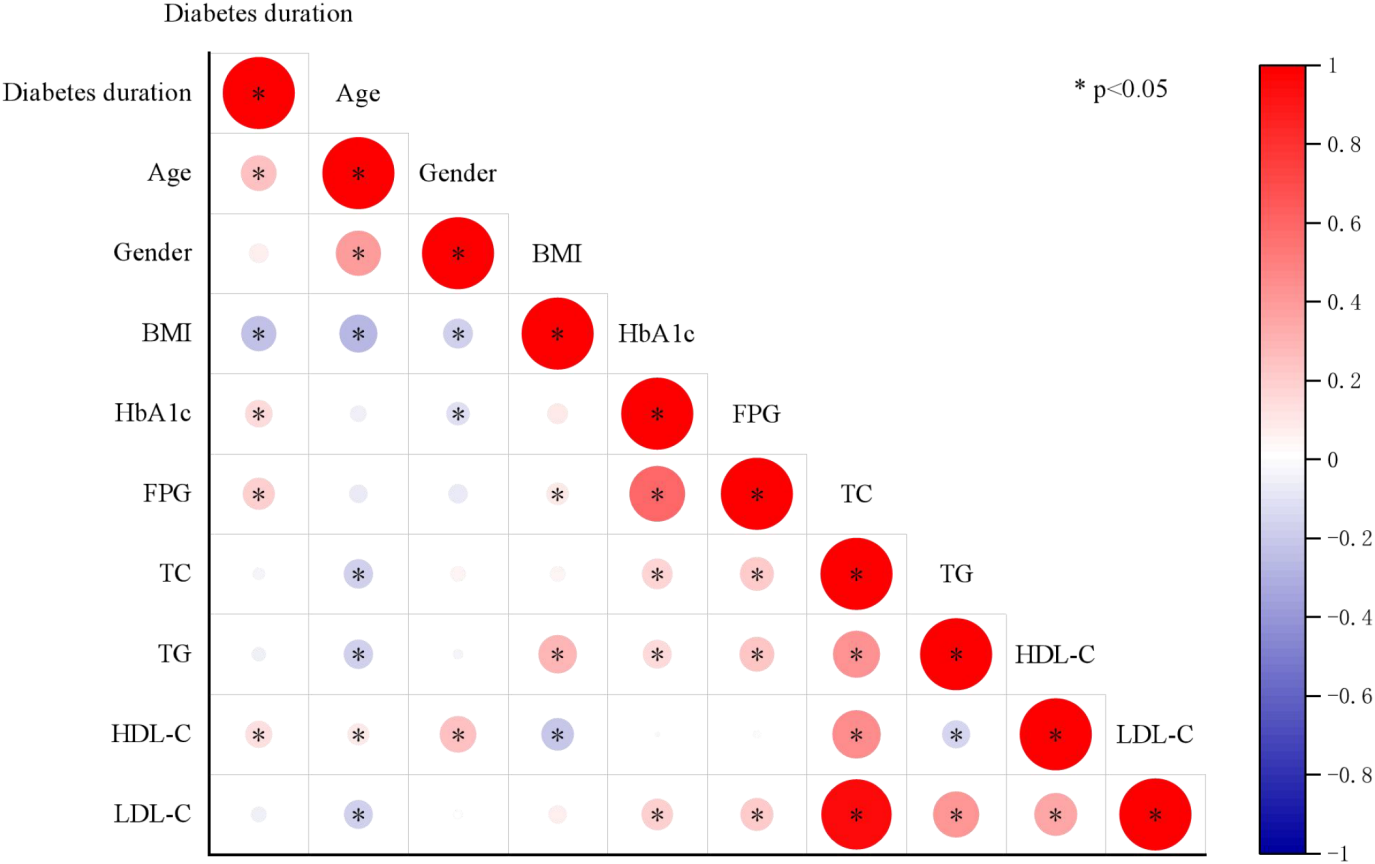
Associations between diabetes duration and traditional cardiovascular disease risk factors.

**Figure 2:**
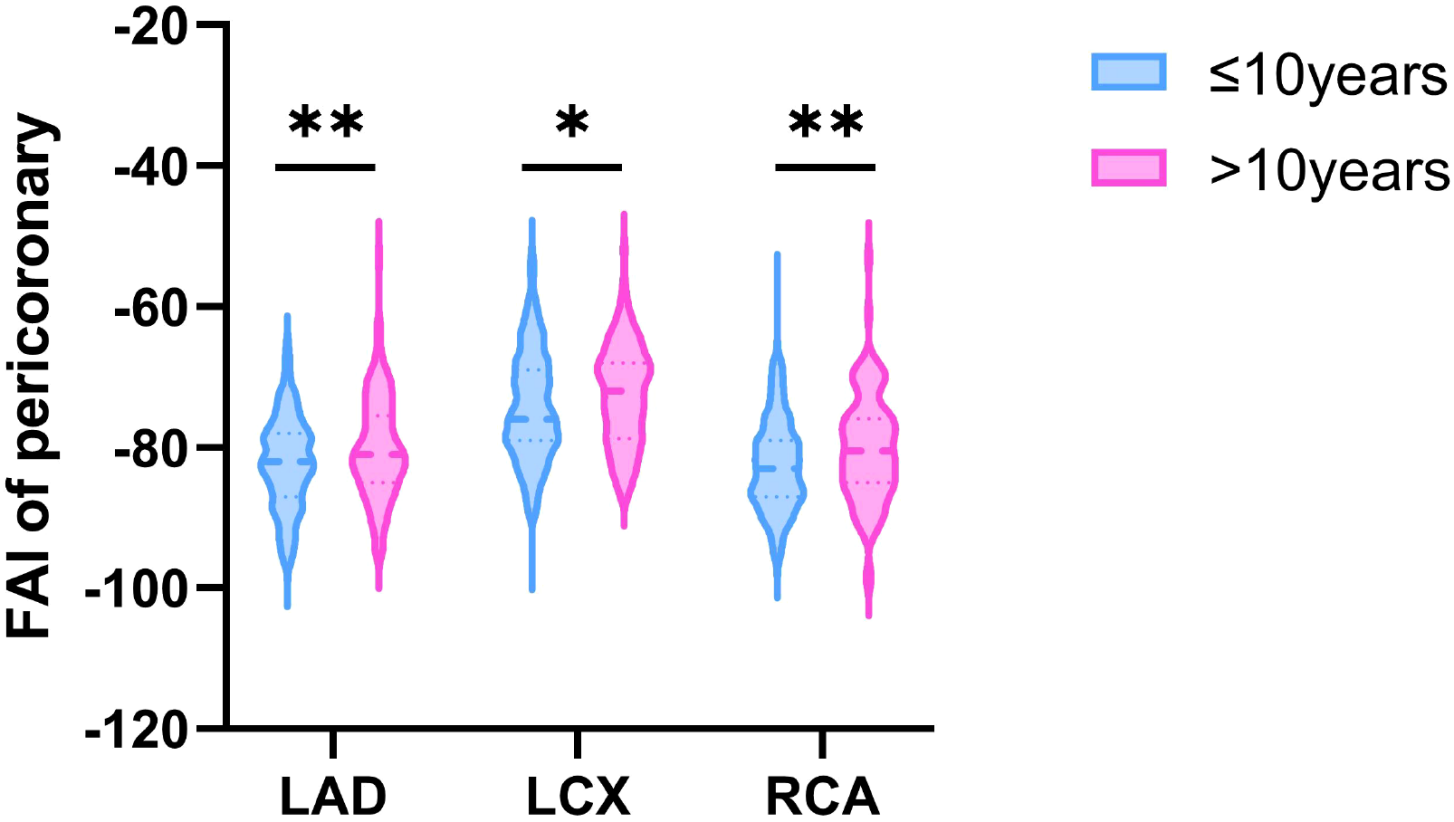
Pericoronary FAI values in three main coronary arteries (356 in the ≤10 years group and 112 in the >10 years group). FAI, fat attenuation index; LAD, left anterior descending artery; LCX, left circumflex artery; RCA, right coronary artery; *P<0.05, **P < 0.01.

The study population was stratified into two groups based on diabetes duration: ≤10 years and >10 years. Baseline demographic and clinical characteristics are summarized in **Table 1**. The median age was 64.0 years, and 40.6% of participants were female. Compared to participants with diabetes duration ≤10 years, those with >10 years of duration were significantly older, had higher levels of fasting plasma glucose (FPG) and HbA1c, and exhibited a higher prevalence of hypertension, as well as more frequent use of antidiabetic and lipid-lowering medications. As illustrated in **Figure 1**, diabetes duration was positively correlated with age, FPG, HbA1c, and HDL-C, and negatively correlated with BMI. Participants with >10 years of diabetes exhibited significantly higher pericoronary FAI values across all three major coronary arteries, as shown in the **Figure 2**.

**Table 1:** Baseline characteristics of the participants.

Parameters	Total (n=468)	Diabetes duration, years		*P*
		≤10(n=356)	>10(n=112)	
Age, years	64.00 (56.75, 72.00)	63.00 (55.00, 72.00)	67.00 (59.00, 73.00)	0.008
Age category, n (%)				0.016
<65	243 (51.92)	196 (55.06)	47 (41.96)	
≥65	225 (48.08)	160 (44.94)	65 (58.04)	
Female, n (%)	190 (40.60)	147 (41.29)	43 (38.39)	0.586
BMI(kg/m2)	25.95 (23.46, 28.69)	26.30 (23.53, 29.22)	25.30 (23.39, 27.71)	0.085
BMI category, n (%)				0.590
<24	145 (30.98)	108 (30.34)	37 (33.04)	
≥24	323 (69.02)	248 (69.66)	75 (66.96)	
Hypertension, n (%)	326 (69.66)	239 (67.13)	87 (77.68)	0.034
Dyslipidaemia, n (%)	210 (44.87)	165 (46.35)	45 (40.18)	0.252
Smoking, n (%)	93 (19.87)	77 (21.63)	16 (14.29)	0.089
Hemoglobin(g/L)	136.00 (124.00, 151.00)	138.00 (124.00, 152.00)	134.00 (120.75, 147.00)	0.122
White blood cells(×109)	6.71 (5.44, 7.71)	6.73 (5.53, 7.77)	6.58 (5.01, 7.60)	0.269
Neutrophils(×109)	4.29 (3.32, 5.30)	4.33 (3.38, 5.29)	4.08 (3.07, 5.34)	0.348
Lymphocytes(×109)	1.69 (1.36, 2.06)	1.72 (1.36, 2.07)	1.65 (1.33, 2.02)	0.280
Platelets(×109)	219.00 (179.75, 258.00)	222.50 (181.00, 261.00)	205.00 (173.00, 242.50)	0.059
HbA1c(%)	7.20 (6.62, 8.30)	7.18 (6.60, 8.10)	7.50 (6.82, 8.62)	0.002
FPG(mmol/L)	7.30 (6.14, 9.33)	7.07 (6.01, 8.91)	8.33 (6.60, 10.37)	<0.001
TC(mmol/L)	4.15 (3.32, 5.03)	4.17 (3.32, 5.03)	4.12 (3.32, 4.95)	1.000
Triglycerides(mmol/L)	1.40 (0.98, 2.11)	1.42 (0.98, 2.11)	1.38 (0.96, 2.10)	0.916
LDL-C(mmol/L)	2.58 (1.94, 3.28)	2.58 (1.95, 3.31)	2.51 (1.89, 3.24)	0.869
HDL-C(mmol/L)	1.12 (0.95, 1.31)	1.11 (0.93, 1.29)	1.17 (1.00, 1.37)	0.100
Antidiabetic agents, n (%)	396 (84.62)	288 (80.90)	108 (96.43)	<0.001
Antihypertensive agents, n (%)	305 (65.17)	227 (63.76)	78 (69.64)	0.255
Antihyperlipidemic agents, n (%)	430 (91.88)	320 (89.89)	110 (98.21)	0.005
LVEF(%)	64.00 (61.00, 67.00)	64.00 (61.00, 67.00)	63.50 (61.00, 66.00)	0.772
CCTA parameters				
CACS	39.99 (0.00, 273.94)	27.12 (0.00, 265.28)	77.37 (0.00, 360.56)	0.099
FAI				
LAD-PCAT (HU)	-81.72 ± 6.78	-82.00 (-87.00, -78.00)	-81.00 (-85.00, -75.50)	0.002
LCX-PCAT (HU)	-75.00 (-79.00, -68.00)	-76.00 (-79.00, -69.00)	-72.00 (-78.25, -68.00)	0.016
RCA-PCAT (HU)	-82.00 (-87.00, -78.00)	-83.00 (-87.00, -79.00)	-80.50 (-85.00, -75.95)	0.002

Continuous data are presented as mean ± standard deviation (SD) or median (25th, 75th percentiles). Categorical data are presented as percentage (%). BMI, body mass index; FPG, fasting plasma glucose; HDL-C, high-density lipoprotein cholesterol; LDL-C, low-density lipoprotein cholesterol; LVEF, left ventricular ejection fraction; CCTA, coronary CT angiography; CACS, coronary artery calcification score; FAI, fat attenuation index; RCA, right coronary artery; LAD, left anterior descending artery; LCX, left circumflex artery; PCAT, pericoronary adipose tissue.

### 3.2 Associations between diabetes duration and pericoronary FAI

**Figure 3:**
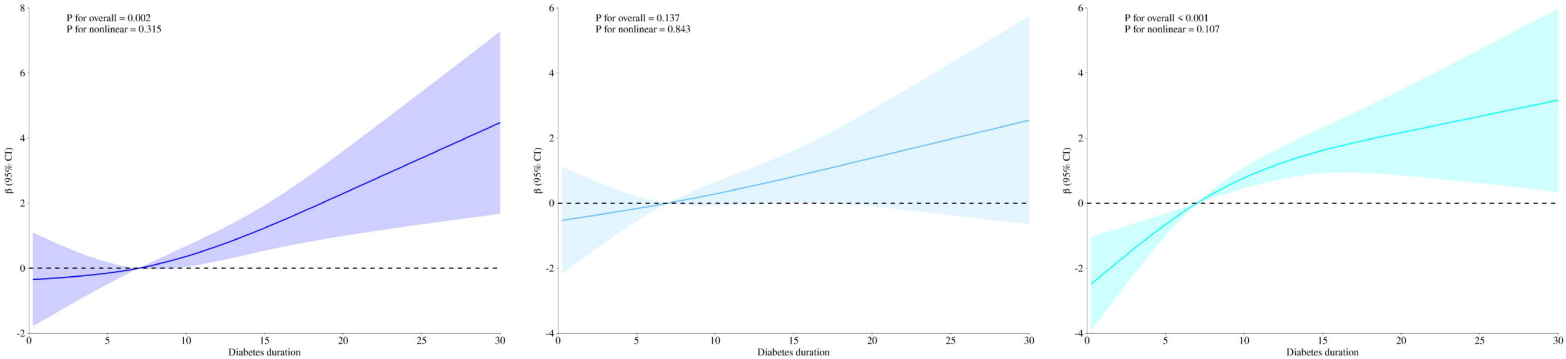
The RCS analysis between diabetes and pericoronary FAI values in three main coronary arteries. FAI, fat attenuation index; LAD, left anterior descending artery; LCX, left circumflex artery; RCA, right coronary artery.

The associations between diabetes duration and pericoronary FAI are detailed in **Table 2**. In both Model 1 and Model 2, a positive association was observed between diabetes duration and pericoronary FAI among patients with T2DM. After full adjustment for covariates in Model 3, diabetes duration remained significantly and positively associated with pericoronary FAI in the LAD artery (β = 0.151, 95% CI: 0.064–0.238, P = 0.001), LCX artery (β = 0.101, 95% CI: 0.001–0.201, P = 0.047), and RCA (β = 0.208, 95% CI: 0.120–0.296, P < 0.001).Sensitivity analysis confirmed the robustness of the findings(**Supplementary Table S2**). RCS models were used to assess potential non-linear associations between diabetes duration and pericoronary FAI, as shown in **Figure 3**. The associations were predominantly linear, with non-significant P-values for non-linearity in the LAD (P = 0.315), LCX (P = 0.843), and RCA (P = 0.107). Stratified analyses revealed heterogeneous associations between diabetes duration and pericoronary FAI across clinical subgroups (**Table 3**). Notably, diabetes duration remained robustly and significantly associated with increased RCA FAI across subgroups, including younger (<65 years: β = 0.31, 95% CI: 0.16–0.47, P < 0.001) and older (≥65 years: β = 0.15, 95% CI: 0.04–0.26, P = 0.007) adults, both males (β = 0.20, 95% CI: 0.08–0.31, P < 0.001) and females (β = 0.23, 95% CI: 0.08–0.37, P = 0.002), and participants with or without hypertension and dyslipidemia. In the LAD, significant associations were observed in males (β = 0.17, 95% CI: 0.05–0.28, P = 0.005), older adults (≥65 years: β = 0.19, 95% CI: 0.08–0.30, P < 0.001), non-smokers (β = 0.18, 95% CI: 0.08–0.27, P < 0.001), individuals with hypertension (β = 0.20, 95% CI: 0.10–0.30, P < 0.001), and those without dyslipidemia (β = 0.19, 95% CI: 0.09–0.30, P < 0.001).For the LCX, significant associations were confined to females (β = 0.16, 95% CI: 0.01–0.32, P = 0.047), older adults (β = 0.12, 95% CI: 0.01–0.24, P = 0.045), non-smokers (β = 0.12, 95% CI: 0.01–0.23, P = 0.028), hypertensive individuals (β = 0.13, 95% CI: 0.02–0.24, P = 0.026), and those without dyslipidemia (β = 0.14, 95% CI: 0.02–0.27, P = 0.028).

**Table 2:** The associations between diabetes duration and e pericoronary FAI.

Variables	β (95%CI)	*P*	Model 2	*P*	Model 3	*P*
	Model 1					
LAD-PCAT						
Diabetes duration	0.140(0.059,0.221)	0.001	0.137(0.052,0.222)	0.002	0.151(0.064,0.238)	0.001
Diabetes duration category						
≤10 years	Reference		Reference		Reference	
>10 years	2.254(0.824,3.685)	0.002	2.105(0.656,3.554)	0.004	2.283(0.820,3.747)	0.002
*P* for trend	0.002		0.004		0.002	
LCX-PCAT						
Diabetes duration	0.113(0.022,0.204)	0.015	0.106(0.010,0.201)	0.030	0.101(0.001,0.201)	0.047
Diabetes duration category						
≤10 years	Reference		Reference		Reference	
>10 years	1.869(0.264,3.474)	0.023	1.652(0.032,3.272)	0.046	1.628(-0.042,3.297)	0.056
*P* for trend	0.023		0.046		0.056	
RCA-PCAT						
Diabetes duration	0.159(0.076,0.241)	<0.001	0.196(0.111,0.281)	<0.001	0.208(0.120,0.296)	<0.001
Diabetes duration category						
≤10 years	Reference		Reference		Reference	
>10 years	2.283(0.833,3.733)	0.002	2.466(1.006,3.927)	0.001	2.513(1.023,4.002)	0.001
*P* for trend	0.002		0.001		0.001	

Model 1 was unadjusted for any covariates. Model 2 adjusted for age, gender, and BMI. Model 3 additionally adjusted for dyslipidemia, smoking, antihyperlipidemic agents, antidiabetic agents, CACS, LVEF, and HbA1c. FAI, fat attenuation index; RCA, right coronary artery; LAD, left anterior descending artery; LCX, left circumflex artery; PCAT, pericoronary adipose tissue.

**Table 3:** Multivariate linear regression analyses between diabetes duration and the pericoronary FAI in different subgroups.

Subgroup	n(%)	LAD-PCAT		LCX-PCAT		RCA-PCAT	
		β(95% CI)	*P*	β(95% CI)	*P*	β(95% CI)	*P*
Age							
<65	243 (51.92)	0.06 (-0.10, 0.21)	0.472	0.02 (-0.17, 0.20)	0.852	0.31 (0.16, 0.47)	<0.001
≥65	225 (48.08)	0.19 (0.08, 0.30)	<0.001	0.12 (0.01, 0.24)	0.045	0.15 (0.04, 0.26)	0.007
Gender							
male	278 (59.40)	0.17 (0.05, 0.28)	0.005	0.07 (-0.06, 0.20)	0.315	0.20 (0.08, 0.31)	<0.001
female	190 (40.60)	0.12 (-0.02, 0.26)	0.088	0.16 (0.01, 0.32)	0.047	0.23 (0.08, 0.37)	0.002
BMI							
<24	145 (30.98)	0.20 (0.06, 0.35)	0.008	0.14 (-0.05, 0.32)	0.148	0.30 (0.13, 0.47)	<0.001
≥24	323 (69.02)	0.14 (0.03, 0.24)	0.015	0.08 (-0.04, 0.21)	0.170	0.16 (0.06, 0.27)	0.003
Smoking							
No	375 (80.13)	0.18 (0.08, 0.27)	<0.001	0.12 (0.01, 0.23)	0.028	0.22 (0.12, 0.31)	<0.001
Yes	93 (19.87)	0.14 (-0.11, 0.40)	0.275	0.06 (-0.19, 0.32)	0.631	0.12 (-0.08, 0.31)	0.232
Hypertension							
No	142 (30.34)	-0.02 (-0.19, 0.16)	0.868	-0.00 (-0.22, 0.22)	0.996	0.37 (0.18, 0.57)	<0.001
Yes	326 (69.66)	0.20 (0.10, 0.30)	<0.001	0.13 (0.02, 0.24)	0.026	0.16 (0.06, 0.26)	0.002
Dyslipidaemia							
No	258 (55.13)	0.19 (0.09, 0.30)	<0.001	0.14 (0.02, 0.27)	0.028	0.16 (0.05, 0.27)	0.004
Yes	210 (44.87)	0.06 (-0.09, 0.20)	0.443	0.02 (-0.14, 0.18)	0.782	0.24 (0.09, 0.38)	0.002

Adjusted for age, gender, BMI, dyslipidemia, smoking, antihyperlipidemic agents, antidiabetic agents, CACS, LVEF, and HbA1c. FAI, fat attenuation index; RCA, right coronary artery; LAD, left anterior descending artery; LCX, left circumflex artery; PCAT, pericoronary adipose tissue.

## 4 Discussion

This study offers new insights into the cardiovascular consequences of diabetes by exploring individual-level pathophysiological quantification. We provide the first quantitative evidence that longer diabetes duration is independently associated with increased coronary inflammation, as assessed by the perivascular FAI, even after adjusting for traditional cardiovascular risk factors.

Vascular inflammation constitutes a pivotal pathogenic mechanism underpinning the progression of atherosclerosis and CVD (35). Pericoronary FAI, an emerging imaging biomarker derived from CCTA, facilitates noninvasive identification of coronary inflammation and serves as a prognostic indicator for major adverse cardiovascular events (36). Data from the CRISP-CT study robustly demonstrated that elevated perivascular FAI is predictive of both all-cause and cardiovascular mortality (37). Chronic, low-grade vascular inflammation represents a fundamental pathophysiological nexus linking diabetes with accelerated atherosclerosis and adverse cardiovascular outcomes. In individuals with T2DM, enhanced activation of the inflammasome complex is evidenced by elevated expression of nucleotide-binding oligomerization domain-like receptor family pyrin domain-containing 3 (NLRP3), concomitant with increased circulating concentrations of the pro-inflammatory cytokines interleukin (IL)-1β and IL-18 (18). Furthermore, NETosis, a unique form of macrophage cell death, is markedly enhanced under hyperglycemic conditions. This process constitutes a direct mechanistic link between atherosclerosis and diabetes, elucidated within the context of inflammatory pathways (38, 39). Prolonged glucotoxicity triggers excessive mitochondrial reactive oxygen species (ROS) generation in perivascular adipocytes, thereby activating the NF-κB/NLRP3 inflammasome signaling cascade (40, 41). Concurrently, accumulation of advanced glycation end products (AGEs) within perivascular adipose tissue (PVAT) promotes sustained transmigration of pro-inflammatory cytokines into the vascular wall through receptor for advanced glycation end products (RAGE)-mediated signaling (42–44).

The Atherosclerosis Risk In Communities (ARIC) Study provided evidence that prolonged duration of diabetes mellitus is associated with an increased incidence of heart failure (45). A large cohort study involving 435,679 participants further indicated that extended diabetes duration correlates with heightened risks of CVD and all-cause mortality (8). Moreover, a prolonged duration of diabetes and elevated levels of coronary inflammation are strongly associated with more diffuse and calcified coronary artery disease, thereby complicating percutaneous coronary intervention and antithrombotic therapy (46). Our study offers the first quantitative evidence demonstrating that prolonged diabetes duration is independently associated with exacerbated coronary inflammation, beyond traditional cardiovascular risk factors.This finding partially elucidates the mechanistic link between diabetes duration and increased cardiovascular disease risk. RCS analysis revealed a near-linear dose–response relationship between diabetes duration and coronary inflammation. This observation, combined with evidence of a sustained age-dependent escalation in the cardiovascular disease burden attributable to high fasting plasma glucose (47), collectively substantiates diabetes duration as a continuous determinant of cardiovascular injury. A population-based investigation demonstrated that the LDL-C threshold associated with increased cardiovascular risk varies according to diabetes duration (48). Accordingly, clinicians should integrate both glycemic control and diabetes duration in cardiovascular risk stratification for individuals with diabetes. Pericoronary FAI, a noninvasive quantitative biomarker for coronary artery inflammation assessed by CCTA, may offer novel insights into risk stratification and facilitate identification of patients who could derive the greatest benefit from targeted anti-inflammatory interventions. Certain pharmacotherapies, including sodium-glucose cotransporter-2 inhibitors (SGLT2i) and glucagon-like peptide-1 receptor agonists (GLP-1RA), have demonstrated potential in attenuating coronary artery inflammation in patients with T2DM (33, 34).

This study offers new insights into the cardiovascular consequences of diabetes by exploring individual-level pathophysiological quantification. Despite these methodological strengths, several limitations warrant careful consideration: (1) The cross-sectional study design inherently precludes causal inference, underscoring the need for future longitudinal investigations to elucidate the temporal dynamics of FAI in relation to disease progression; (2) External validation is imperative due to the single-center cohort design, which may limit generalizability. Therefore, multi-center studies involving more diverse ethnic and clinical backgrounds are warranted to validate and extend our results; (3) Although adjustments were made for numerous confounding variables, residual confounding from unmeasured factors(such as technical heterogeneity) may nevertheless influence the observed associations.

## 5 Conclusion

Prolonged diabetes duration is independently linked to heightened coronary inflammation, as quantified by pericoronary FAI. These findings provide mechanistic insights into the elevated CVD risk associated with long-standing diabetes. Pericoronary FAI may serve as a novel imaging biomarker for cardiovascular risk stratification and may aid in identifying individuals most likely to benefit from targeted anti-inflammatory therapies.

## Data Availability

The raw data supporting the conclusions of this article will be made available by the authors, without undue reservation.
