# Correction: Quantifying the link: coronary artery inflammation via CCTA-derived fat attenuation index and its association with diabetes duration

**DOI:** 10.3389/fendo.2025.1768980

**Published:** 2026-01-02

**Authors:** Frontiers Production Office

**Affiliations:** Frontiers Media SA, Lausanne, Switzerland

**Keywords:** cardiovascular disease, high fasting plasma glucose, pericoronary fat attenuation index, coronary artery inflammation, diabetes duration

In the **Abstract**, the third sentence of the **Materials and methods** section was written as “Multivariablee association between diabetes duration and pericoronary FAI”. This has been corrected to read: “Multivariable linear regression and subgroup analyses were performed to evaluate the association between diabetes duration and pericoronary FAI.”

The original version of this article has been updated.

