# Supplementary material for: Quantifying the link: coronary artery inflammation via CCTA-derived fat attenuation index and its association with diabetes duration
Source: Front Endocrinol (Lausanne). 2025 Dec 4;16:1671949. doi: 10.3389/fendo.2025.1671949 (PMC12711490; doi:10.3389/fendo.2025.1671949)
Supplement: Supplementary file 1 [file DataSheet1.docx]

| **Table S1** Collinearity statistics in the primary analysis | | |
| --- | --- | --- |
| **Variable** | **Tolerance** | **VIF** |
| Diabetes duration | 0.827 | 1.210 |
| Age | 0.683 | 1.464 |
| Gender | 0.687 | 1.456 |
| BMI | 0.871 | 1.148 |
| Smoking status | 0.822 | 1.217 |
| Dyslipidemia | 0.931 | 1074 |
| HbA1c | 0.916 | 1.092 |
| LVEF | 0.970 | 1.031 |
| CACS | 0.965 | 1.037 |
| Antidiabetic agents | 0.909 | 1.100 |
| Antihyperlipidemic agents | 0.907 | 1.103 |

| **Table S2** Sensitivity analysis | | |
| --- | --- | --- |
|  | **β (95%CI)** | ***P*** |
| **LAD-PCAT** |  |  |
| Diabetes duration | 0.150(0.056,0.244) | 0.002 |
| Diabetes duration category |  |  |
| ≤10 years | Reference |  |
| >10 years | 2.353(0.791,3.916) | 0.003 |
| *P* for trend | 0.003 |  |
| **LCX-PCAT** |  |  |
| Diabetes duration | 0.119(0.011,0.227) | 0.030 |
| Diabetes duration category |  |  |
| ≤10 years | Reference |  |
| >10 years | 1.992(0.201,3.782) | 0.029 |
| *P* for trend | 0.029 |  |
| **RCA-PCAT** |  |  |
| Diabetes duration | 0.216(0.120,0.311) | <0.001 |
| Diabetes duration category |  |  |
| ≤10 years | Reference |  |
| >10 years | 2.592(0.996,4.187) | 0.002 |
| *P* for trend | 0.002 |  |

Adjusted for age, gender, BMI, dyslipidemia, smoking, stain, PCSK9i, SGLT2i, GLP1-RA, insulin, metformin, sulfonylurea, α-glycosidase inhibitor, CACS, LVEF, and HbA1c.

| **Table S3** Collinearity statistics in the sensitivity analysis | | |
| --- | --- | --- |
| **Variable** | **Tolerance** | **VIF** |
| Diabetes duration | 0.697 | 1.435 |
| Age | 0.633 | 1.581 |
| Gender | 0.678 | 1.475 |
| BMI | 0.825 | 1.213 |
| Smoking status | 0.816 | 1.225 |
| Dyslipidemia | 0.827 | 1.209 |
| HbA1c | 0.830 | 1.205 |
| LVEF | 0.953 | 1.049 |
| CACS | 0.959 | 1.042 |
| Stain | 0.896 | 1.116 |
| PCSK9i | 0.805 | 1.243 |
| SGLT2i | 0.914 | 1.095 |
| GLP1-RA | 0.824 | 1.214 |
| Insulin | 0.704 | 1.420 |
| Metformin | 0.954 | 1.049 |
| Sulfonylurea | 0.938 | 1.066 |
| α-glycosidase inhibitor | 0.902 | 1.109 |
